# Different evasion strategies in multiple myeloma

**DOI:** 10.3389/fimmu.2024.1346211

**Published:** 2024-02-23

**Authors:** Chaofan Wang, Wanting Wang, Moran Wang, Jun Deng, Chunyan Sun, Yu Hu, Shanshan Luo

**Affiliations:** Institute of Hematology, Union Hospital, Tongji Medical College, Huazhong University of Science and Technology, Wuhan, China

**Keywords:** multiple myeloma, evasion, extrinsic mechanism, intrinsic mechanism, immunology

## Abstract

Multiple myeloma is the second most common malignant hematologic malignancy which evolved different strategies for immune escape from the host immune surveillance and drug resistance, including uncontrolled proliferation of malignant plasma cells in the bone marrow, genetic mutations, or deletion of tumor antigens to escape from special targets and so. Therefore, it is a big challenge to efficiently treat multiple myeloma patients. Despite recent applications of immunomodulatory drugs (IMiDS), protease inhibitors (PI), targeted monoclonal antibodies (mAb), and even hematopoietic stem cell transplantation (HSCT), it remains hardly curable. Summarizing the possible evasion strategies can help design specific drugs for multiple myeloma treatment. This review aims to provide an integrative overview of the intrinsic and extrinsic evasion mechanisms as well as recently discovered microbiota utilized by multiple myeloma for immune evasion and drug resistance, hopefully providing a theoretical basis for the rational design of specific immunotherapies or drug combinations to prevent the uncontrolled proliferation of MM, overcome drug resistance and improve patient survival.

## 1 Introduction

**Figure 1:**
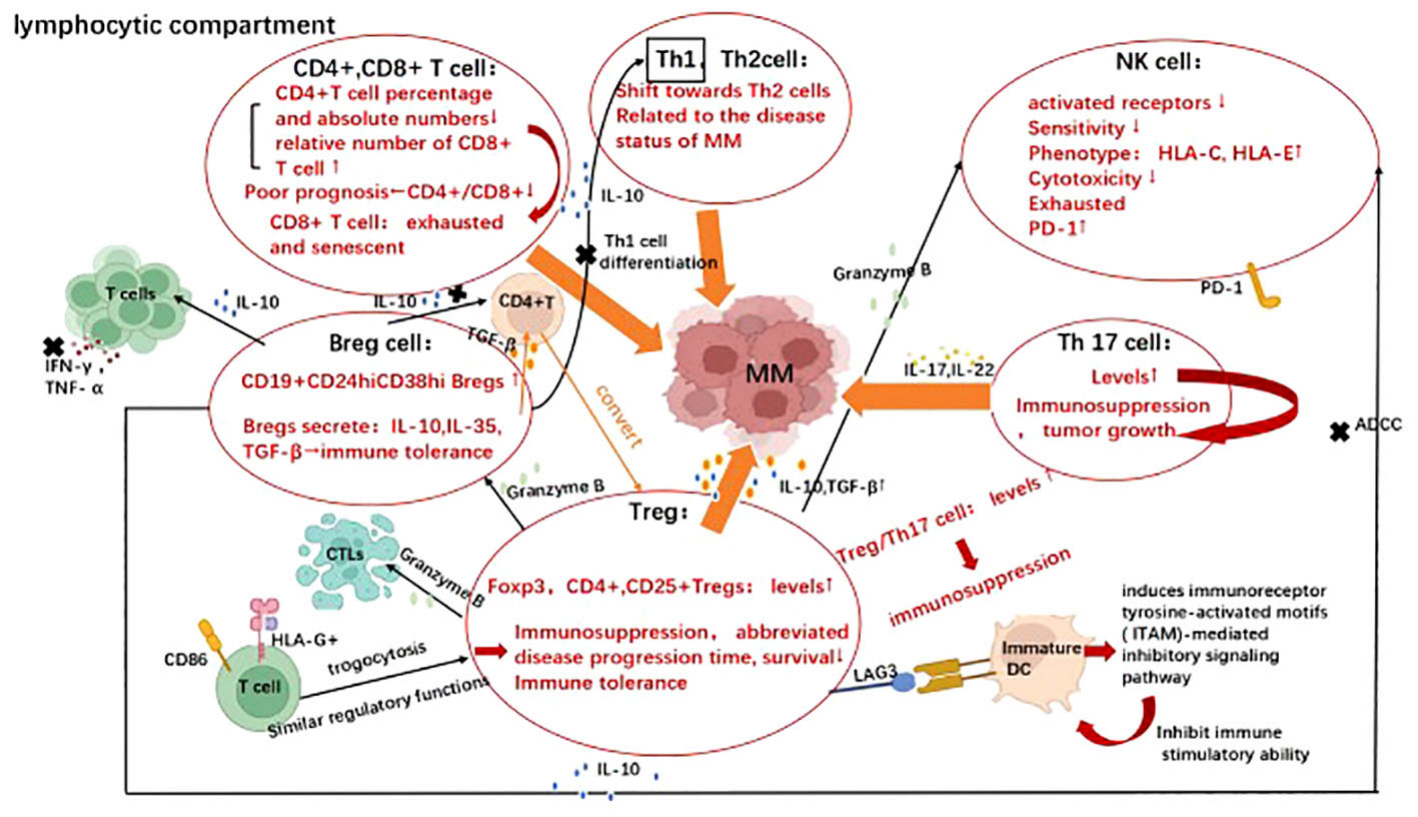
Evasion strategies represent the cross-talk between multiple myeloma with various lymphocytes. Immune evasion mechanisms utilized by multiple myeloma involve both the lymphoid and myeloid compartments. Image illustrates the evasion strategies employed by various lymphocytes in multiple myeloma, including effector T cells (CD4+, CD8+ T cells), T helper cells (Th1, Th2, Th17, Treg), B cells and NK cells. Their cross-talk with multiple myeloma in the presence of virous cytokines promote disease progression and facilitate immune escape. Created with Biorender.com.

**Figure 2:**
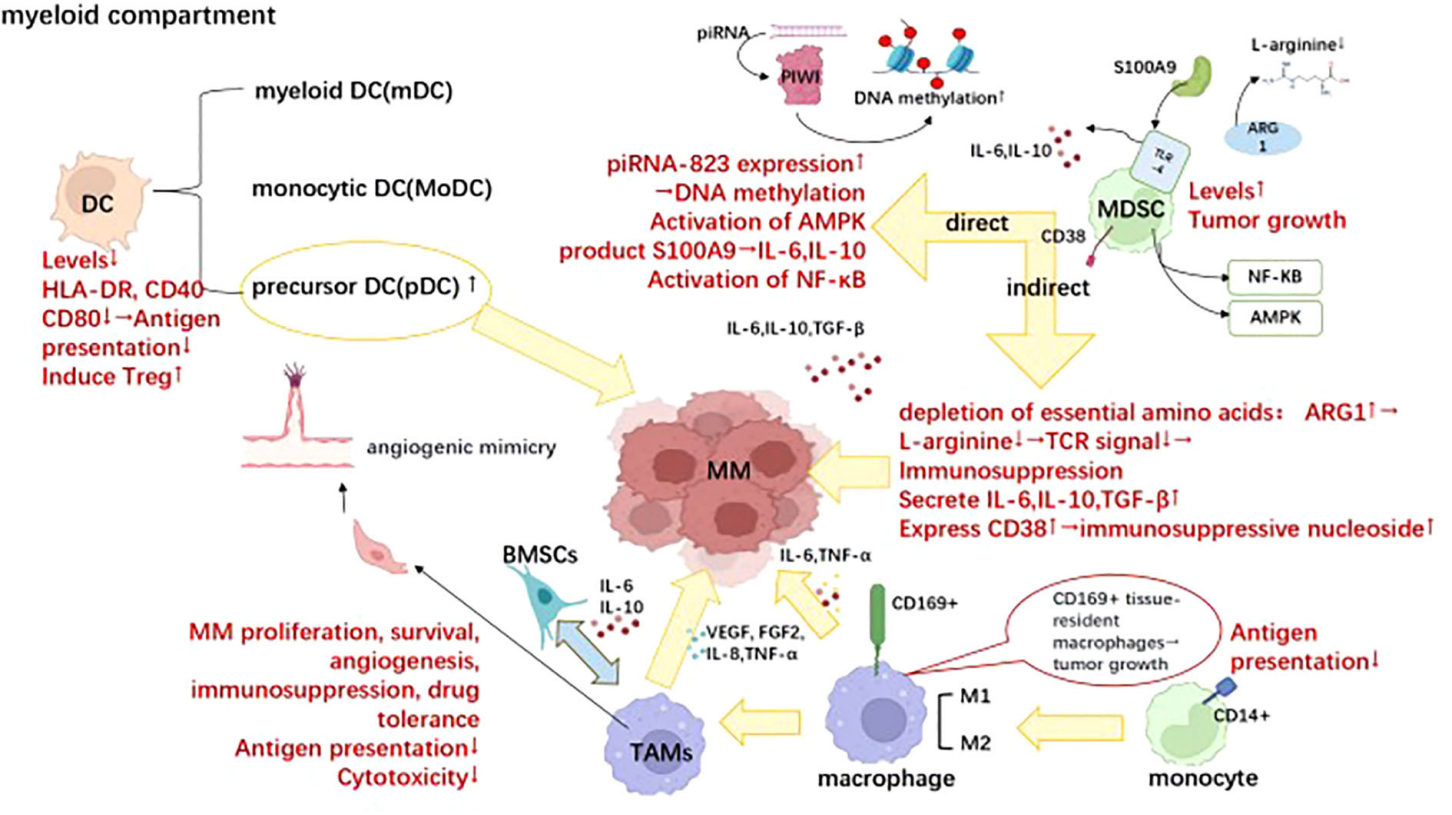
Differential cellular components within the bone marrow microenvironment involved in immune evasion of MM. Dendritic cells (DCs), myeloid-derived suppressor cells (MDSCs), and tumor-associated macrophages (TAMs) are crucial cellular components within the bone marrow microenvironment involved in the immune evasion of multiple myeloma, which plays pivotal roles in the progression of multiple myeloma. Briefly, the reduction in normal DC numbers is accompanied by a decline in antigen presentation ability; however, the accumulation of precursor DC cells drives the progression of multiple myeloma. MDSCs exert immunosuppressive effects and promote tumor growth through both direct and indirect pathways. TAMs exhibit diminished immune cytotoxicity and antigen presentation ability while promoting tumor survival and expansion through cytokine release such as IL-6 and IL-10. Moreover, they can induce vascular mimicry formation and release VEGF, thereby facilitating tumor angiogenesis. This figure was. Created with Biorender.com.

Multiple myeloma (MM) is a malignant tumor developed from the progressive growth and proliferation of clonal transformed plasma cells (PCs) present in the bone marrow (BM) (1–3). MM is the second most common hematologic malignancy. In the general statistics of cancer, the incidence of MM is 1%, and increases with age, accounting for 13% of hematological malignancies (4, 5). 50% of diagnosed cases are older than 75 years, but rare in those younger than 40 years (6, 7). MM is characterized by a multi-step evolutionary pathway, starting with the early asymptomatic stage, defined as monoclonal γ disease of unknown significance (MGUS). Among those, 1% of cases generally evolved into the dominant disease each year, through an intermediate stage called “smoldering” MM (SMM) (8). Dominant MM promotes terminal organ dysfunction with the accumulation of clonal PCs in myeloma, which is clinically diagnosed as anemia, osteolytic disease, hypercalcemia, and kidney damage (1, 9). Modern myeloma treatment has dramatically improved patient outcomes, even with 5-year overall survival (OS) nearly doubling (10). However, despite these improvements, MM still remains incurable (11–13). On the one hand, similarly to other types of cancers, MM is a genetically complex and heterogeneous disease resulting from multiple genomic events that trigger tumor development and progression. Primary genomic events are usually divided into hyperdiploid (HRD) and non-HRD subtypes. Most non-HRD tumors harbor typical translocations affecting the genes encoding immunoglobulin (Ig)heavy chains (IgH) — mainly t(4;14), t(6;14), t(11;14), t(14;16) and t(14;20), while those patients lacking immunoglobulin heavy chain(IgH) translocations have hyperdiploid chromosome number (HMM) with trisomies of chromosomes 3,5,7,9,11,15,19 and 21. Secondary events provide a fitness advantage to a particular subclone over other populations and are required for tumor development and progression. For example, most copy-number variations (CNV), translocations involving the MYC gene family, and somatic mutations affect mitogen-activated protein kinase (MAPK), nuclear factor-κB (NF-κB), and DNA-repair pathways (14). On the other hand, the biological progress of MM from MGUS to dominant disease indicates that the bidirectional interaction between myeloma cells with BM auxiliary cells and extracellular matrix induces autocrine and paracrine signaling. Furthermore, tumor cells utilize secreted soluble factors such as cytokines, growth factors, chemokines,etc. as well as cell-cell adhesion to transform the bone marrow microenvironment into an immunosuppressive milieu (15, 16). The immunosuppressive environment not only supports the tumor progression but also promotes its escape. Next, it cannot be neglected that the influence of the microbiota on host immunity is also essential. Researchers reported that the microbiota plays a potential role in the pathogenesis of hematologic malignancies such as MM by influencing inflammatory signaling pathways and host metabolism. Meanwhile, microbial metabolites also influence the immunity and prognosis of patients with myeloma and hematological malignancies (17). Therefore, the focus of our current research should not only be limited to the tumor metabolism and progression but also be extended to the diverse evasion mechanisms and drug resistance. This review aims to provide an integrative overview of the intrinsic and extrinsic mechanisms (**Figure 1**, **2**) as well as recently discovered microbiota utilized by MM for immune evasion and drug resistance, hopefully providing a theoretical basis for the rational design of specific drugs or combinations to prevent the immune escape of MM, to overcome drug resistance of MM, thereby aiming to improve patient survival rates.

## 2 Intrinsic mechanism

### 2.1 Immunoediting

**Figure 3:**
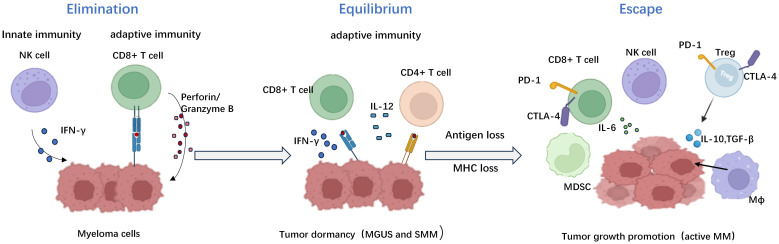
The three distinct phases of immune editing in multiple myeloma. The immune editing phases in multiple myeloma include the elimination phase, equilibrium phase, and escape phase respectively. During the elimination phase, tumor cell eradication is mediated by both humoral and cellular immunity. The equilibrium phase represents a critical transitional period characterized by continuous sculpting of tumors under immune pressure, ultimately leading to dormancy breakage and subsequent relapse and dissemination. The escape phase signifies the proliferation of multiple myeloma as tumors employ diverse mechanisms to evade immune surveillance. Created with Biorender.com.

“Immune editing” unifies the immune system’s dual host defense and tumor sculpting capabilities (18). Dunn, G. P et al. reported that elimination, equilibrium, and escape processes lead to cancer immune editing (19 ). (**Figure 3**) During the elimination phase, immune surveillance preserves intracellular homeostasis by intrinsic and adaptive immunity, thereby continually identifying and eliminating altered cells malignantly (18). Experimental data performed by comparison of tumor growth and metastasis between wild-type and immunodeficient mouse models showed that Natural Killer(NK) cells, CD8+ T cells, and their effector molecules perforin and Interferon-γ(IFN-γ) play key roles in myeloma recognition and elimination (19). Any tumor cell variants that survive the elimination process will enter dynamic equilibrium. In this phase, the immune system is likely silent, and keeps residual tumor cells in a dormant functional state, eventually resuming growth in the form of recurrent primary tumors or distant metastases. Stronger evidence about the existence of an equilibrium phase came from primary tumorigenesis experiments reported by Catherine M. Koebel, which showed that immunocompetent mice treated with low doses of a carcinogen [3 ‘-methylcholanthraxime (MCA)] were able to “hide” cancer cells for long periods. When T cells and IFN-γ were depleted, tumors rapidly appeared at the original MCA injection site in half of the mice. Tumor cells separated from these lesions had high immunogenicity. Further analysis shows that the adaptive immunity in this model of mice, such as interleukin 12 (IL - 12), IFN - γ, CD4+, CD8+ T cells, instead of innate immune displayed ability to maintain the balance of the occult tumor cells. This observation mechanistically distinguishes equilibrium from elimination, as the latter exhibits an obligate requirement for innate and adaptive immunity (20). For example, in the development of MM, asymptomatic PC dyscrasias (MGUS and SMM) may represent the equilibrium phase (21). Experiments performed by Das. R showed that xenografted PC from MGUS patients showed progressive growth in immunodeficient humanized mice, suggesting that the disease latency was correlated with host immune conditions (22). What’s more, Dhodapkar et al. also showed that the lack of anti-SOX2 (an embryonic stem cell antigen)T cells was significantly associated with the progression to active MM in patients with MGUS or asymptomatic MM. It highlights the importance of tumor-specific T cells in preventing disease progression (23). The last phase is called escape. The surviving tumor variants escaping immune detection begin to expand uncontrollably which leads to clinically observed malignant disease that, if left uncontrolled, can result in death (24).

### 2.2 Reduced immune recognition of tumor antigens

The reduction in immune recognition is mainly due to the absence or reduced expression of tumor antigens or defects in processing and presentation. Under immune selection, in order to survive, cancer cells undergo immunogenic mutations, thereby prompting loss of tumor antigenicity (18). A case of irreversible B cell-maturation antigen(BCMA) loss was reported in a patient with MM who was enrolled in the KarMMa trial (NCT03361748) and progressed after anti-BCMA chimeric antigen receptor (CAR) T-cell therapy. Furthermore, the researchers identified the selection of tumor necrosis factor receptor superfamily 17(TNFRSF17) homozygous deletion clones (BCMA) as a potential immune escape strategy. In addition, they identified TNFRSF17 heterozygous deletion or 16 monosomy in 37 of 168 MM patients, including 28 of 33 hyperhaploid MM patients who had not previously received BCMA-targeted therapy, suggesting that TNFRSF17 heterozygous deletion at baseline could theoretically be a risk factor for BCMA loss after immunotherapy (25). Furthermore, during the CAR-T therapy, the selection for a BCMA double allele loss clone after initial CAR-T cell infusion by deletion of one allele and a mutation produced an early termination codon on the second allele. This deletion resulted in a lack of proliferation of CAR-T cells after the second infusion, which was also reflected by the lack of soluble BCMA in the patient’s serum (26). In a recent clinical trial, the anti-G protein–coupled receptor, class C group 5 member D(GPRC5D) CAR T-cell therapy has exhibited promising clinical efficacy and manageable safety profiles in patients with relapsed/refractory MM, thus providing an additional alternative for individuals who have experienced disease progression or developed resistance to anti-BCMA CAR-T treatment (27). Meanwhile, two rare cases of relapsed refractory MM patients that were initially CD38 positive but spontaneously converted to CD38 negative were reported (28), suggesting CD38 is also involved in antigen deletion. In terms of antigen processing and presentation, it was found that transporters associated with antigen processing(TAP) expression were related to the presentation of major histocompatibility complex(MHC) class I molecules. One of the myeloma cell lines (NCI-H929) included in the study showed reduced TAP peptide transport activity. Although TAP expression levels were comparable in the cell lines, NCI-H929 cell surface expression levels of MHC class I molecules were significantly reduced. When IFN-γ was supplied to NCI-H929 cells, TAP expression and peptide transport activity were upregulated (29). Similarly, the expression of proteasome subunits and peptide transport proteins was lower, while the level of chaperone proteins was higher in MM samples (29). Changes in the antigen processing-presentation machinery in the pathogenesis sequence may allow transformed plasma cells to evade immune surveillance (30).

### 2.3 Genomic alterations

Intraclonal heterogeneity is present at all stages of tumor progression, which is through the transformation of high-risk (HR)-SMM into MM, a typical disease feature (31). For example, translocations involving immunoglobulin (Ig) loci and chromosome 13 monosomes are common cytogenetic findings in multiple myeloma, and similar translocations exist in both MGUS and MM, including t(4;14)(p16.3;q32) and t(14;16)(q32;q23). In addition, chromosome 13 monosomy is common in MGUS, but unlikely to play a dominant role in the progression of MGUS to MM (32). Experts found that the GABA receptor-associated protein(GABARAP) locus is located at chr17p13.1, a region that is missing in high-risk (HR) MM with poor prognosis, and that low GABARAP levels were significantly associated with shorter event-free survival(EFS) even for MM patients without del17p. This mechanism may explain why patients with del17p HR MM had poor clinical outcomes, thus suggesting that new therapies that restore GABARAP may help to improve the outcome of MM patients (33). In MM progression, some extramedullary myeloma cells expressing the tumor necrosis factor -related apoptosis-inducing ligand(TRAIL) gene trigger aberrant activation of TRAIL receptors on the surface of T cells, therefore inhibit proximal T cell receptor(TCR)signaling and suppress human T cells activation and proliferation (34). Moreover, t(14;16) with a mutation in B-Raf proto-oncogene(BRAF) exits in a small subset of patients with newly diagnosed multiple myeloma, this mutation has been observed to be associated with an increased incidence of advanced refractory disease and unfavorable outcomes (35). A prospective single-arm, open-label, multicenter phase 2 trial evaluated the efficacy and safety of combined BRAF/mitogen-activated protein kinase(MEK) inhibition (using encorafenib and binimetinib) in patients with relapsed/refractory multiple myeloma (RRMM) carrying the BRAFV600E mutation (NCT02834364). This study represents the first prospective clinical trial to demonstrate the remarkable efficacy of BRAF/MEK combination inhibition in patients with RRMM harboring the BRAFV600E mutation, thus exemplifying a successful implementation of targeted precision medicine for this malignancy. Although the initiating event of myeloma is clonal, the subsequent driving lesions usually occur in subclones of cells, thereby promoting disease progression through a Darwinian selection process. Therefore, understanding the co-evolution process of clones in their local microenvironment is essential for the further therapeutic manipulation of this event (36).

## 3 Extrinsic mechanism

### 3.1 lymphocytic compartment

#### 3.1.1 T cells

Effectively immunoreactive T cells in tumor microenvironment(TME) are required to successfully eliminate tumor cells. Defects in T cell activity and tissue distribution have been reported in MM patients (37–39). Immunoreactive T cells normally encompass helper T cells and effector T cells. Among the effector T cells, the percentage and absolute number of CD4+ cells were significantly decreased in MM patients. In contrast, the relative number of CD8+ cells increased only slightly (40). Low CD4+ T-cell counts and CD4/CD8 ratios at diagnosis were independent predictors of poor prognosis in MM patients (41). The effector T cell therapies, including CAR-T, bispecific T cell Engager (BiTE), and antibody-drug conjugate(ADC), have been applied in clinical practice. CAR-T therapies targeting BCMA, GP5CRD, Signaling lymphocytic activation molecule family member 7(SLAMF7), CD38 etc. along with BITE and ADC technologies have demonstrated remarkable efficacy in clinical settings, leading to breakthrough therapeutic outcomes (42). T helper cells(Th) mainly comprise Th1, Th2, Th17, regulatory cell(Treg), and follicular helper T (Tfh) cells. Furthermore, elevated peripheral blood Th1/Th2 ratios have also been reported in patients with primary and refractory MM (43). In addition, the equilibrium between Treg and Th17 cells is pivotal for effective anti-tumor immune response. An increased Treg/Th17 ratio results in a bias toward immunosuppression. The experts showed that serum levels of interleukin(IL)-17, IL-22 as well as the frequency of Th17 cells produced by bone marrow and peripheral blood mononuclear cells were significantly increased in myeloma patients compared with healthy donors, suggesting that Th17-associated pro-inflammatory cytokines may be present in MM BM microenvironment and may regulate MM cell growth as well as immune response. Then, they further observed that IL-17 promotes myeloma cell growth, colonization, and adhesion to bone marrow stromal cells (BMSC) through blinding to IL-17 receptor, as well as increased the *in vivo* growth in murine xenotransplantation model of human MM (44).Likewise, the highly significant statistical relationship between the IL-17 in bone marrow and osteoclast formation also strongly supports the promotion of IL-17 to osteolytic disease (45). Next, it is confirmed that IL-22 promotes signal transducer and activator of transcription 3(STAT-3) phosphorylation, cell growth in IL-22RA1+(IL-22 receptor) MM cells. Moreover, the treatment of IL-22RA1+MM cells with IL-22 increases both cell growth through p38 mitogen activated protein kinase (MAPK)activation and resistance to drug-induced cell death through up-regulation of myeloid leukemia 1 (Mcl-1) expression and inhibition of drug-induced caspase-3 activation (46). The population of CD4+ CD25+ Treg cells is elevated in the peripheral blood of MM patients, and the upregulation of transcription factor 3 (Foxp3) in CD4+ CD25+ Treg cells is correlated with their immunosuppressive activity (47). In addition, increased Treg cell count in MM patients is pertaining to abbreviated disease progression time and diminished survival (48). Treg cells derived from MM patients exhibit potent suppressor activity against antigen-presenting cells (APCs) and T cell proliferation. Detailedly, it can be classified into three basic “modes of action”: inhibition by secretion of suppressive cytokines, inhibition by cytolysis, and inhibition by regulation of dendritic cell (DC) maturation or function (49). Compared to healthy individuals, patients with MM exhibit significantly elevated levels of transforming growth factor-beta (TGF-β) and interleukin-10 (IL-10) secreted by regulatory T cells (Tregs), indicating an enhanced suppressive activity. Notably, Treg cells also secrete granzyme B, resulting in the apoptosis of cytotoxic T lymphocytes (CTLs), B cells, and natural killer cells (NK cells) (50–52) Regarding the immunomodulatory effects mediated by Treg cells on DC maturation and function, the binding of lymphocyte activation gene-3(LAG3) to immature DC-expressing MHC class II molecules induces immunoreceptor tyrosine-activated motifs (ITAM)-mediated inhibitory signaling pathway, thereby inhibiting DC maturation and immune stimulatory capacity (53–56). In addition, CD86 and human leukocyte antigen G (HLA-G) are common antigens acquired by T cells from malignant plasma cells (trogocytosis) and HLA-G+ T cells, which have regulatory functions similar to those of natural Treg cells, providing another novel mechanism for evading effective immune surveillance utilized by MM cells (57). Tregs-related therapy has also made a great breakthrough in MM treatment. For example, *in vivo* removal of Treg in mice with established multiple myeloma induced effective CD8 T-cell and NK cell-mediated immune responses, leading to complete and stable remission of MM. This preclinical *in vivo* study suggests that Treg is an attractive target for treating MM (58). In addition, since SLAMF7 is highly expressed on immunosuppressive CD8+CD28-CD57+ Treg cells in patients with MM, anti-SLAMF 7 treatment efficiently cleared suppressive T cells from the peripheral blood of MM patients. It is suggested that anti-SLAMF7 antibodies can enhance the anti-tumor immune responses in MM patients (59). Changes in the Treg compartment identified during MM progression are essential for improving our understanding of clinical stability of MGUS and MM pathology, and further for improving the clinical diagnosis, prognosis, and therapeutic implications of MM with potential implications (60).

#### 3.1.2 B cells

*B regulatory(Breg) cell* is an immunosuppressive cell that supports immune tolerance through the production of interleukin 10 (IL-10), IL-35, and transforming growth factor β (TGF-β). Recent studies have shown that different inflammatory environments formed during the MM progression induce different Breg cell phenotypes (61). For example, in the bone marrow microenvironment of MM patients, CD19+CD24hiCD38hi Bregs expression is increased (62). This type of CD19+CD24hiCD38hi B cells can inhibit CD4+CD25 - T cell proliferation and suppress th1 cell differentiation, thereby inhibiting the release of the pro-inflammatory cytokines, such as IFN-γ and TNF- α by T cells. This inhibition is partially mediated by the production of interleukin-10 (IL-10) (63). The addition of CD80 and CD86 monoclonal antibodies can reverse such an inhibition (64). Furthermore, the bone marrow-derived CD19+CD24highCD38high Breg population abrogates NK cell-mediated antibody-dependent cell mediated cytotoxicity (ADCC) against MM by producing IL-10 (65). Next, Bregs also convert CD4+ T cells into activated Tregs by secreting transforming growth factor-beta (TGF-β), which in turn inhibits T cell proliferation and promotes tumor metastasis (66, 67). Therapeutically, the effective treatment with daratumumab is accompanied by the eradication of Bregs in addition to its anti-myeloma effects (62).

#### 3.1.3 NK cells

NK cell is a type of lymphocyte characteristic with CD3-CD56+, exerting antitumor effects not only through direct killing and triggering ADCC, but also indirectly through induction of cytokines release, such as granzyme, IFN-γ, and TNF-α (68–70). NK cells express a range of receptors, especially on the cell surface, including receptors for inhibition, activation, adhesion, and cytokines. When in contact with interacting cells via surface-expressed receptors, various signals may be transduced simultaneously, in turn determining whether NK cells will be activated (69, 71–73). Interestingly, the expression of activated receptors such as natural killer group 2D receptor(NKG2D), natural killer p30 (NKp30) and natural killer p46(NKp46) on bone marrow natural killer cells was found to be suppressed in patients with MM compared to healthy volunteers (74, 75). Notably, tumor cells evade immune surveillance by down-regulating NK cells’ sensitivity and upregulating human leukocyte antigen C(HLA-C) and human leukocyte antigen E (HLA-E) expression (69, 72, 76, 77). Proteasome inhibitors (Bortezomib and Carfilzomib) can induce NK cell-mediated killing of MM cells by down-regulating MHC-I expression on the surface of MM cells (78, 79). Also, NK cells from MM patients express programmed cell death protein 1(PD-1), whereas normal NK cells do not. The combination of PD-1 and programmed death-ligand 1(PD- L1) down-regulates the effect of NK cells mediated elimination of MM. It was proved that the novel anti-PD-1 antibody CT-011 enhances the killing of autologous primary MM cells by human NK cells. Notably, a phase 2 clinical trial of CT-011 in combination with lenalidomide for the treatment of MM patients warrants consideration (80). In MM, but not MGUS, myeloma cells shed soluble MHC class I-related chain A (sMICA), resulting in downregulation of NKG2D expression, impaired lymphocytotoxicity, and resistance to NKG2D-mediated killing (81–83). These findings revealed that changes in the NKG2D path were associated with progression from MGUS to MM, and increased the likelihood of application of anti-MICA monoclonal antibodies to treat these diseases (83). Experts also found that MM, by promoting the upregulation of CXC-chemokine receptor 3(CXCR3) ligands and downregulating of CXC-chemokine receptor 12 ligands(CXCL12), driven NK cells outside of the bone marrow, thereby impairing the antitumor immune responses at the primary site (84). In terms of therapies, developing CAR-NK cells is an exciting approach for MM treatment. Due to the isolation of NK cells and the absence of MHC restriction for tumor cell eradication, various approaches were also developed for the clinical application of NK cell-based therapies, such as bi-specific antibodies specific to NK cells (85). Moreover, remarkable advancements have been achieved in the management of MM by means of ex *vivo* expansion and activation of NK cells, followed by autologous and haploidentical infusion. (NCT03019640).

### 3.2 Myeloid compartment

#### 3.2.1 Myeloid-derived suppressive cells

MDSCs are mainly divided into two subtypes: granulocytic myeloid-derived suppressive cells(G-MDSCs): CD11b+CD14−CD15+/CD66b+; monocytic myeloid-derived suppressive cells(M-MDSCs): CD14+CD15−HLA- DRlo/– (86). MDSCs are a heterogeneous group of immature hematopoietic cells that play a role in MM cell survival, tumor progression, and immunosuppression (87). In return, MM can also promote the accumulation and activation of MDSCs, thus forming a vicious cycle (88). The regulatory mechanisms managing MDSCs’ acceleration of myeloma progression are primarily composed of direct and indirect methods. In relation to the direct mechanism, Lisha Ai et al. demonstrated that G-MDSCs induced piRNA-823 expression, which in turn facilitated DNA methylation and incorporated stem cell characteristics into MM cells (89). As observed by experts, the activation of adenosine monophosphateactivated kinase (AMPK) partially mediates the tumor-promoting effect of MDSC in mice (90). Additionally, the production of S100A9, a calcium-binding protein that stimulates the secretion of TNF-α, IL-6, and IL-10 through toll-like receptor 4(TLR4)-mediated autocrine signaling, by MDSCs in the tumor microenvironment (TME) attracts myeloma cells (91) and fosters their growth by activating the canonical nuclear factor-kappa B(NF-κB) pathway (92). MDSCs indirectly affect MM cells’ survival through the modulation of nutrient availability, secretion of soluble factors, and the expression of cell surface inhibitory molecules. MDSCs enhance the expression of enzymes that contribute to the depletion of essential amino acids, such as L-arginine, required for effective TCR-mediated signaling (93, 94). Kavita Ramji et al. investigated that arginase 1(ARG 1) expression in myeloid cells strongly suppressed antigen-induced adoptive transfer of T cell proliferation in a murine MM Vκ*MYC model, accompanied by a systemic decrease in L-arginine levels. Furthermore, they found T cell proliferation was restored and Vκ*MYC tumor progression was significantly delayed when they knocked out ARG1 in MM-bearing mice or applied ARG inhibitors (95). In addition,multiple soluble factors,and cytokines, such as IL-10, IL-6, TGF-β, CD40-CD40 ligand, and IFN-γ, contribute to the immune-suppressive activity of MDSC in the TME, tipping the scales in favor of Tregs (96, 97). Purposefully, the expression of CD38 on MDSCs play a crucial role in the discontinuous multi-cellular pathway of adenosine (Ado) (98), a highly represented immune-suppressive nucleoside in the TME of MM patients (99). The expression of immunecheckpoint (ICP)/ICP-L plays a role in the impairment of anti-myeloma immune responses. As previously demonstrated, PD-L1 is expressed by BM MDSC in all disease states and potentially contributes to the suppression of anti-myeloma activity by PD1+ effector cells (100, 101). To break free from this detrimental cycle, studies have shown that macrophage migration inhibitory factor(MIF) present in the MM microenvironment induces bone marrow cells to express CD84, resulting in upregulation of PD-L1 expression on the surface of MDSCs (102). The presented data demonstrate that galectin-1, derived from MM, could mediate the tumor-promoting effect of M-MDSCs through its interaction with CD304 on M-MDSCs and contribute to tumor progression post-ASCT (103). These newly discovered molecules are expected to be used to as specific markers to target MDSCs for the treatment of MM.

#### 3.2.2 Dendritic cells

Dendritic cells(DCs) have an antigen-presenting role and bridge innate and adaptive immunity. It is divided into three main subtypes plasma cell-like DC precursor cells (pDCs), myeloid DCs (mDCs), and monocytic DCs (MoDCs) (104). The absolute numbers of circulating MoDCs, mDCs, and pDCs were significantly lower in MM patients than those in healthy subjects, both at diagnosis and during the malignant process (105). In addition, during MGUS-MM progression, the aggregation of mDCs and pDCs in the bone marrow was correlated with the tumor load (106). Myeloma peripheral blood DCs(PBDCs) mature with reduced expression of leukocyte antigen (HLA-DR), CD40, and CD80 antigens. Reduced expression of MHC class II molecules and costimulatory molecules is responsible for the defective induction of T cell proliferation. In contrast, Mo-DCs and PBDCs from healthy controls are potent T-cell immunostimulatory factors. Both *in vitro* and *in vivo* models of human MM have shown that pDC promotes MM cell growth, survival, chemotaxis, and drug resistance (bortezomib, lenalidomide). Their findings identify an important role of pDCs in MM pathogenesis and provide a basis for targeting pDC-MM interactions with cytosine-phosphate-guanosine (CpG) oligonucleotides (ODNs), as a therapeutic strategy to improve MM patient prognosis (107). The effect of DCs-mediated induction of Treg depends on the nature of the DCs maturation stimulus with inflammatory cytokine-treated DCs (Cyt-DCs) being the most effective Treg inducer. DC-induced Treg from both healthy donors and MM patients functionally and effectively suppresses T-cell responses (108). DC vaccination has developed from generation to generation over the years. To maximize the therapeutic potential of DC-based therapies, new approaches for the treatment of MM that combine dendritic cell-based immunotherapy with conventional therapies, including chemotherapy, immunomodulatory drugs (IMiDs; Lenalidomide and pomalidomide), and immune checkpoint inhibitors (ICIs; PD-L1) have been proposed (103). In addition, many cytokines were shown to modulate DCs functions. For example, IL-6 inhibits CD34+ DC progenitor cell colony growth, thus converting CD34+ cells from DCs to CD14+ CD1a+ monocytes that exert powerful phagocytosis capacity, but have low antigen presentation capacity. In neutralization experiments, this effect is reversed by anti-IL-6 antibodies (105). In addition, TGF-β1 and IL-10 secreted by myeloma have a role in inhibiting CD80/CD86 upregulation during DC maturation (105, 109). Anti-TGF-β1 antibody, anti-IL-10 antibody or IL-12, IFN-γ infusion can neutralize such an inhibitory effect due to CD80/CD86 upregulation (110). In a recent randomized phase II trial (NCT02728102), experts evaluated the impact of the DC/MM fusion vaccine in combination with maintenance therapy. Although no significant increase in disease response was observed, a notable association with a substantial rise in circulating multiple myeloma-reactive lymphocytes indicative of tumor-specific immunity was identified. These results also lay the foundation for the future development of immunotherapeutic modalities to restore the immune function of DCs, thereby inhibiting tumor growth in MM patients (111).

#### 3.2.3 Macrophages/monocytes

CD14+ monocytes in the MM microenvironment present defective antigen presentation ability due to intracellular HLA-DR accumulation, thus leading to the accumulation of MM and the suppression of T-cell activation (112). Tumor-associated macrophages (TAMs) are derived from circulating monocytes, and their high plasticity and heterogeneity depend on the microenvironment (113, 114). TAMs can be classified into anti-tumor type M1 and tumor-promoting type M2. Recently, CD169+ tissue-resident macrophages were reported to promote early dissemination of MM by secreting IL-6 and TNF-α (115, 116). M2-type components can promote MM proliferation, survival, angiogenesis, as well as immunosuppression, and drug tolerance (117–122). Mechanically, TAMs interact with bone marrow stromal cells(BMSCs) to acquire the function of secreting IL-6 and IL-10, poorly secreting IL-12 and TNF-α, which are suitable for tumor growth (123). In addition, TAMs are not only a rich source of pro-angiogenic cytokines and growth factors’ production such as vascular endothelial growth factor(VEGF), TNF-a, IL-8, and fibroblast growth factor 2(FGF-2), but also assume a vascular endothelial cell-like phenotype after structural activation by VEGF and an essential fibroblast growth factor (bFGF) through “ angiogenic mimicry” contribution to the myeloma vascular network (124). Unlike healthy tissue, macrophages from MM patients cannot phagocytose tumor cells, deliver antigens, and stimulate adaptive immune responses (125, 126). Notably, in MM the expression of IL-10 by TMAs inhibits the activation of cytotoxic T cells and downregulates the expression of crucial cytotoxic T cell factors, including granzyme B and IFN-γ (125–128). Anti-CD47 antibodies have now been found to significantly enhance the ability of macrophages to phagocytose human MM cell lines *in vitro* (129). Finally, TAMs protect myeloma cell lines and primary myeloma cells from spontaneous and chemotherapeutic drug-induced apoptosis by attenuating caspase-dependent activation and cleavage of apoptotic signals as well as P-selectin glycoprotein ligand 1(PSGL-1)/selectins and intercellular adhesion molecule 1(ICAM-1)/CD18, a pattern of cell-cell contact (117, 130). Macrophage treatments are in infancy, aiming for removing and reprogramming of these invalid TAMs. Wang et al. showed that MM tumor load was significantly reduced *in vivo* after removing macrophages in optimally selected transgenic mice (126). Similarly, the IKAROS family zinc finger 1 (IKZF1)- interferon regulatory factor 4(IRF4)/interferon regulatory factor 5(IRF5) axis and the Regulator of G Protein Signaling 12 (RGS12) protein induce myeloma-associated macrophage phenotype switch and functionally skew towards the M1 phenotype, thereby resulting in inhibition of MM growth (131, 132). These results suggest that targeting IKZF1 and RGS12 may provide a novel strategy for reprogramming myeloma-associated macrophages for better MM treatment (133, 134). Moreover, cellular inhibitors of apoptosis proteins (cIAP) 1 and cIAP 2 are amplified in approximately 3% of cancers, rendering them potential therapeutic targets across various malignancies. Small molecule IAP antagonists, such as LCL161, have entered clinical trials due to their capacity to induce TNF-mediated cancer cell apoptosis. Remarkably, we observed robust *in vivo* anti-myeloma activity of LCL161 in a transgenic mouse model of bone marrow cancer and RRMM patients. This effect was not solely attributed to direct induction of tumor cell death but rather stemmed from the upregulation of interferon(IFN) signaling by the tumor cells themselves, leading to potent inflammatory responses that activated macrophages and resulted in phagocytosis of the tumor cells. Treatment with LCL161 conferred long-term protection against tumors in the mouse model and induced regression of tumors in select mice. These findings underscore the profound preclinical anti-myeloma effects exerted by LCL161 (135).

#### 3.2.4 Tumor microenvironment

**Figure 4:**
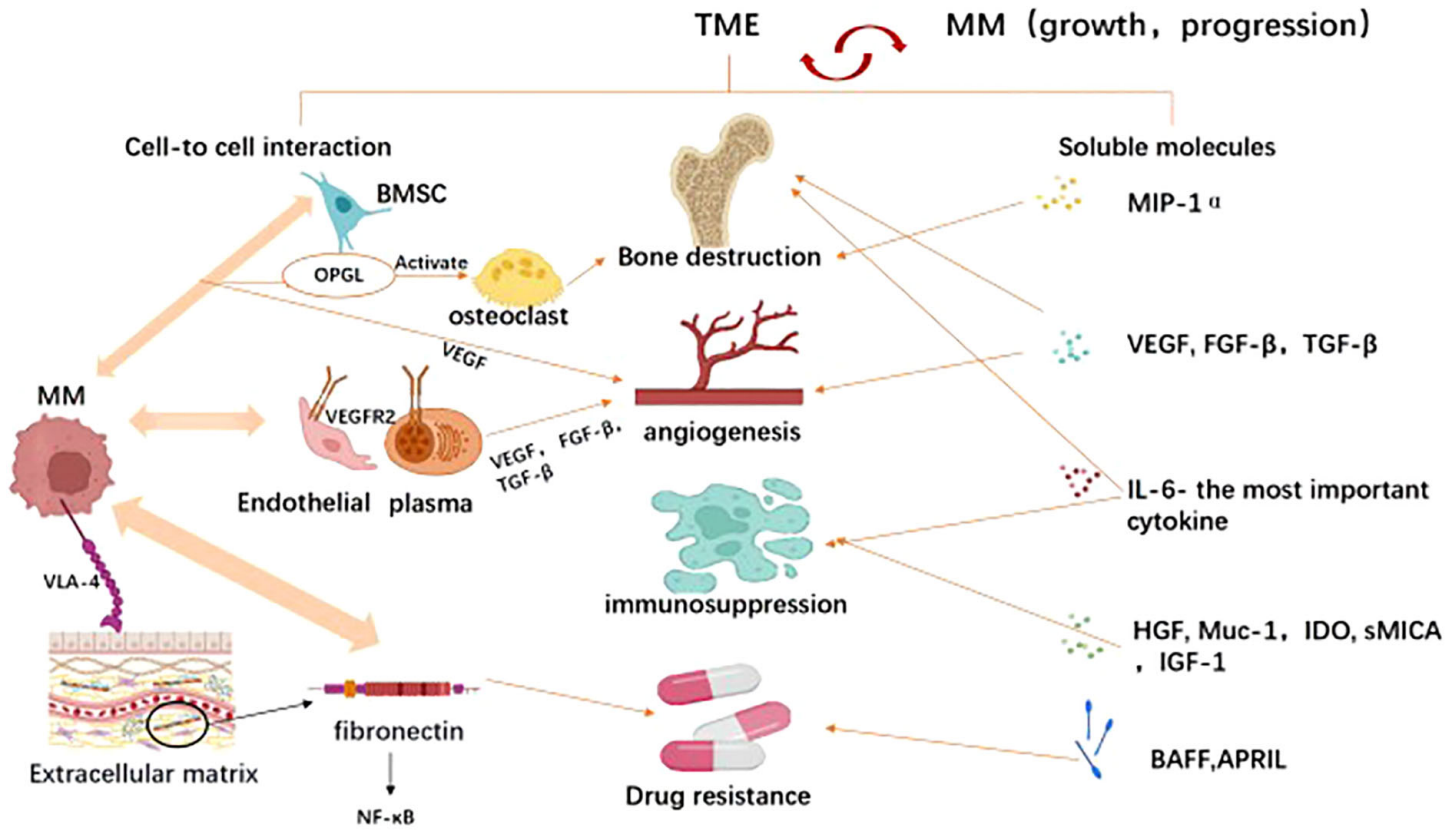
The tumor microenvironment (TME) mediated tumor immune evasion for MM promotion and progression. TME comprises hematopoietic cells, stromal cells, and extracellular matrix components, which interact with multiple myeloma to facilitate its survival and progression. As illustrated In Image, the tumor microenvironment promotes tumor immune evasion through two mechanisms: cell-cell interactions and long-distance effects mediated by released soluble factors. Through cellular interactions, tumor cells engage with endothelial cells, plasma cells, osteoclasts, bone marrow stromal cells, and extracellular matrix components to enhance tumor angiogenesis, induce bone damage, suppress immunity, and foster drug resistance. Similarly, soluble factors in the microenvironment such as MIP-1α (macrophage inflammatory protein), VEGF (vascular endothelial growth factor), FGF-β (fibroblast growth factor-beta), TGF-β (transforming growth factor-beta), IL-6 (interleukin-6), BAFF (B-cell activating factor belonging to the TNF family), and HGF (hepatocyte growth factor) also exert similar roles. Created with Biorender.com.

Tumor microenvironment(TME) is a sophisticated network containing hematopoietic or mesenchymal origin cells, extracellular matrix(ECM), and chemokines, growth factors as well as other soluble molecules, which are produced or influenced by the intercellular compartments (136). TME plays an important role in MM development and progression, triggering bone destruction, angiogenesis, immunosuppression, and drug resistance through cell-cell interactions and soluble molecules (137). (**Figure 4**) For example, osteoprotegerin (OPG) and its ligand (OPGL) are main factors in bone resorption. OPGL stimulates osteoclast differentiation and activation, whereas OPG inhibits these processes. The experts found that myeloma cells react with bone marrow stromal cells in a cell-to-cell interaction, resulting in overexpression of OPGL and downregulation of OPG expression in myeloma patients, which is in turn involved in the pathogenesis of MM-induced bone disease (138). In addition, the chemokine macrophage inflammatory protein-1α (MIP-1α) also promotes osteolytic lesions’ development (139). The process of angiogenesis is primarily triggered by factors released by bone marrow stromal cells and myeloma cells. Among them, vascular endothelial growth factor(VEGF) is the main molecule that promotes angiogenesis (140, 141). VEGF-a is an important isoform of VEGF because its receptor VRGFR2 is overexpressed on endothelial and plasma cells of patients with MM (142, 143). They interact with each other to induce not only VEGF but also other pro-angiogenic factors such as basic fibroblast growth factor(FGF-β), TGF-β (144). Among the soluble molecules, IL-6 is the most important cytokine involved in the pathogenesis and disease progression of MM. It was reported that IL-6 promoted myeloma proliferation, prevented cells from apoptosis, enhanced bone destruction, most importantly, triggered immunosuppression (45, 145, 146). The immunosuppressive effect is mainly reflected in that IL-6 can reduce the ability of DC cells to present specific antigens to autologous T cells, thus inhibiting T activation (105). Recent evidence suggested that several other molecules, including HGF, membrane-bound mucin glycoprotein (Muc-1), indoleamine 2,3-dioxygenase (IDO), as well as soluble primary histocompatibility complex class I -related chain A (sMICA), can also be found in the tumor microenvironment. High levels of hepatocyte growth factor (HGF) in the serum and bone marrow of MM patients correlate with a poor prognosis and HGF also indirectly inhibits antigen-induced T-cell activation (147, 148). Meanwhile, the membrane-bound mucin glycoprotein (Muc-1), indoleamine 2,3-dioxygenase (IDO) and soluble primary histocompatibility complex class I -related chain A (sMICA) suppress innate and adaptive immunity and play an essential role in tumor prognosis (149–151). Drug-resistance induced by the microenvironment can be seen as the sum of resistance caused by direct cell adhesion(CAM-DR) and resistance caused by soluble molecules(SM-DR) (152). As for SM-DR, both TNF superfamily members BAFF (B cell-activating factor of the TNF family) and APRIL (a proliferation-inducing ligand) stimulate the survival of tumor cells, thus conferring their role in drug resistance (153). Besides, it is confirmed that high levels of IGF-1 cytokine are associated with bortezomib resistance (154). Compared with SM-DR, CAM-DR is more important (155). MM connects to the extracellular matrix through activated VLA-4(known as integrin α4β1), while VLA-4 binds to fibronectin to stimulate NF-κB signaling pathway activation, promoting pro-survival signaling and CAM-DR (156–158). Emerging reports have shown that solid interactions between clonal plasma cells and the surrounding bone marrow microenvironment contribute to the pathophysiology of MM. Based on these resistance strategies identified in MM, one can have the rationale for the preclinical evaluation of new therapeutic approaches to target MM clones and the BM environments for better suppression and prevention of MM disease progression (159).

## 4 Microbiota mechanism

**Figure 5:**
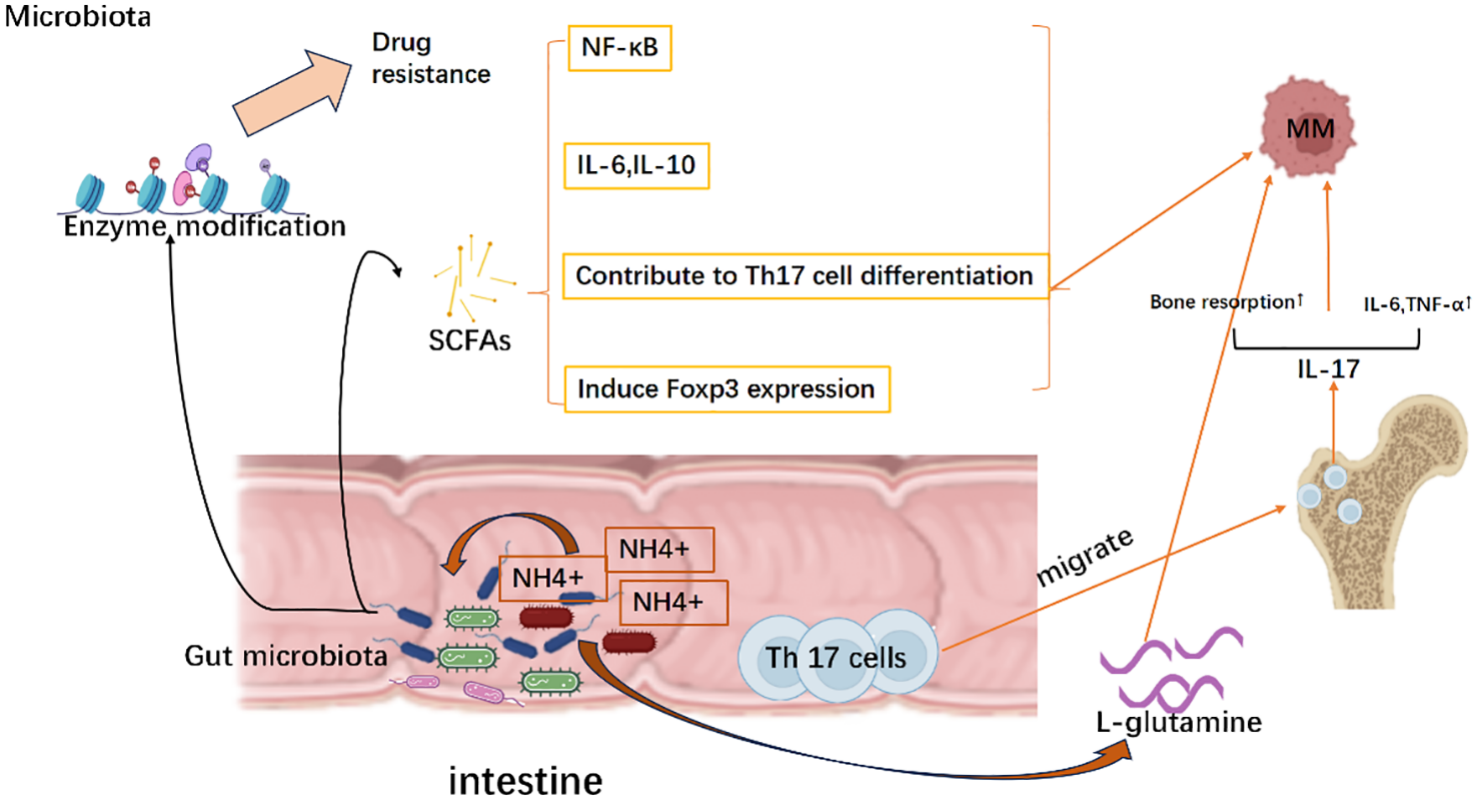
Evasion strategies of MM mediated by the microbiota. Mechanically, the microbiota facilitates the release of short-chain fatty acids (SCFA), which subsequently stimulates the production of interleukin-6 (IL-6) and interleukin-10 (IL-10). This activation further triggers the nuclear factor kappa B (NK-κB) signaling pathway activation and induces Foxp3 expression as well as differentiation of Th17 cells, thereby establishing a tumor microenvironment. Meanwhile, as NH4+ scavengers, microbiota can utilize NH4+ to generate L-glutamine that promotes the growth of multiple myeloma. Simultaneously, gut-resident Th17 cells migrate to the bone marrow and secrete cytokines such as IL-17 and IL-6, leading to bone destruction and accelerating disease progression of multiple myeloma. Moreover, gut microbiota can modify drug-metabolizing enzymes, which in turn affects treatment responses by promoting the development of drug resistance in multiple myeloma. Created with Biorender.com.

The microbiota plays an essential role in maintain the immune homeostasis, dysfunction of which can cause different types of diseases all the time. More attention is being paid to the complexity of host-microbial interactions. Recently, emerging evidence of a potential association between the microbiota and multiple myeloma has been reported. (**Figure 5**) Symbiotic microorganisms can not only influence local immunity through metabolites and interaction with innate immune cells, but also influence immune cells outside the gut to regulate systemic immune (17).Despite great efforts paid to understand the immune escape of MM, little is known about the microbiota as a novel risk mechanism (160).Here we discuss the effects of microbial metabolites such as short-chain fatty acids(SCFA), L-glutamine, microbiota mediated stimulation of immune cells for MM escape and the investigations of microbial-mediated drug resistance mechanisms in MM. Deep understanding of the microbiota can help to illustrate-some existing drug resistance.

### 4.1 Short-chain fatty acids

Short-chain fatty acids(SCFAs) are bacterial compounds responsible for ion uptake, intestinal motility, and regulation of the immune response. SCFAs can trigger nuclear factor-kappa B(NF-kB) activation and pro-inflammatory cytokines production, including IL-6 and TNF-α, contribute to Th17 cell differentiation. Conversely, SCFAs may also elevate IL-10 levels and induce FoxP3 expression, leading to the differentiation of CD4+ T cells into immunosuppressive types (Treg) (161, 162). Researchers found that butyrate, an SCFA produced by *Eubacterium halii* or *Faecalibacterium prausnitzii*, was associated with negative microscopic residual lesions following the treatment of myeloma (163) Close correlation of SCFA with MM treatment is a biomarker of progression-free survival, suggesting a link between microbial traits and patient outcomes (163).

### 4.2 L-glutamine

Jian et al. reported that opportunistic nitrogen-cycling bacteria like *Klebsiella* and *Streptococcus* enriched the intestinal flora of MM patients. MM cells likely utilized the host intestinal microbiota as NH4+ recyclers to produce the L-glutamine for tumor growth. This finding also justifies the possibility of using fluoro glutamine as a PET tracer in MM (160, 164).

### 4.3 Th17 cells

The imbalanced crosstalk between host and gut flora will lead to immune hyperactivation and Th17 cell amplification. According to the published data using the transgenic Vk*MYC mice, *Prevotella heparinolytica* was proven to promote the development of Th17 cells, which then colonized in the gut and migrated into the bone marrow, advanced MM from asymptomatic to symptomatic form, thereby accelerating the course of MM. The absence of IL-17 or disruption of the microbiome in Vk*MYC mice delayed the progression of MM (165–167). Moreover, treatment of Vk*MYC mice with antibodies against IL-17, IL-17RA, and IL-5 reduced the aggregation of Th17 cells and eosinophils in the bone marrow, which in turn prevented the MM progression (167).

### 4.4 Enzymatic modifications and integrality

Gut microbiota can positively or negatively affect the efficacy of some anti-cancer agents *in vivo* or *in vitro* (**Table 1**), mostly by enzymatic modifications (173). For example, the cytotoxicity of cladribine, gemcitabine, as well as anti-cancer antibiotics (such as doxorubicin) was reduced by bacteria through enzymatic modifications of these drugs. On the contrary, after the enzymatic modification, the cytotoxicity of fludarabine and 6-mercaptopurine was increased (168). Meanwhile, effective drug response also requires a complete microbiota. An *in vivo* murine model confirmed that cyclophosphamide is an important agent for the treatment of hematologic malignancies. In this model, authors showed that cyclophosphamide, by altering the composition of the microbiota in the small intestine and inducing the translocation of selected species of Gram+ bacteria to secondary lymphoid organs, stimulated the production of a specific “pathogenic” T-helper 17 (pTh17) cell subset and Th1 immune responses. These modified T-cell responses in turn efficiently controlled tumor growth. However, when these Gram+ bacteria were treated by antibiotics, tumor-bearing mice showed reduced pTh17 responses, thereby tumors became resistant to cyclophosphamide. The antitumor effect of cyclophosphamide was partially restored by the adoptive transfer of pTh17 cells (169). These results suggest that intact microbial populations to some extent can reduce drug resistance.

**Table 1:** Effects of microbiota on anti-cancer agents.

ANTI-CANCER DRUGS	MICROBIOTA	OVERCOME	MECHANISM
Cladribine,gemcitabine,doxorubicin,Idarubicin,etoposide, mitoxantrone	*E. Coli* or *L. welshimeri* (168)	Decreased efficacy	Enzymatic modification
Fludarabine, 6-Mercaptopurine	*E. coli* (168)	Enhanced efficacy	
Cyclophosphamide	*Lactobacillus species,Segmented filamentous bacteria* (169)	Enhanced efficacy	Promoted Th17 and Th1 cell response
	*Enterococcus hirae,Barnesiella intestinihominis* (170)	Enhanced efficacy	increased CD8/Treg ratio and infiltration of interferon-g-producing gd T cells
Oxaliplatin	*Fusobacterium nucleatum* (171)	Decreased efficacy	Modulation autophagy pathway and inhibit tumor cell apoptosis
PD-1/PD-L1 inhibitor	*F. prausnitzii* *A. muciniphila* (172).	Enhanced efficacy	SCFAs boost CD8+ T cell effector functions by modifying their cellular metabolism

## 5 Conclusion and perspectives

Multiple myeloma (MM) is a malignant tumor developed from the progressive growth and proliferation of clonal transformed plasma cells (PCs) present in the bone marrow (BM). In order to survive, MM evolved different strategies for immune escape and drug resistance. As reviewed in the work, the intrinsic and extrinsic mechanisms as well as recently discovered microbiota typical mechanisms were utilized for MM initiation and progression. As for the intrinsic mechanism, MM sustains a balance between disease dormancy and progression through immune editing, undergoing genetic mutations that facilitate clonal evolution to evade the immune system under selective pressure. Additionally, MM generates an immunosuppressive tumor microenvironment (TME) externally, leading to an upsurge in the population of tumor-suppressive cells, including TAMs, Tregs, Bregs, and MDSCs. This, in turn, results in a decline of effector cytotoxic cells, concomitant with weakened humoral and cytotoxic immunity. Microbiota presents a novel approach that alters the immune microenvironment through its metabolites, such as short-chain fatty acids (SCFAs) and L-glutamine, interacting with Th17 cells to prompt their expansion and potentiate anti-tumor immune functions. Furthermore, the gut microbiota can contribute to drug resistance by modifying enzymes, resulting in refractory and relapse cases in patients with multiple myeloma.

Despite the significant advancements in multiple myeloma management achieved through the utilization of treatment options to address immune evasion, a state of equilibrium is reached between residual disease and the host immune system. This equilibrium leads to either sustained remission or disease relapse. The convergent evolution leading to antigen escape represents a pivotal mechanism for clinical relapse in multiple myeloma (MM). Recent studies have demonstrated that immunotherapies targeting BCMA and GPRC5D can dynamically reshape the antigenicity and clonal landscape of MM cells, reflecting immune editing under targeted immune pressure. Clones harboring extracellular domain mutations in BCMA or GPRC5D may exhibit a higher frequency under longitudinal therapeutic selection pressure from repeated T cell engagers (TCEs) administration compared to chimeric antigen receptor–expressing T cells (CAR T cells) therapy (which is administered only once). These findings challenge the prevailing notion that post-treatment loss of BCMA antigen is infrequent and underscore the intricate mechanisms of antigen escape beyond TNFRSF17 allelic loss.

Typically, the progression from MGUS to MM is exceptionally complex, with intra-tumoral heterogeneity accompanying the entire process. To date, the majority of studies have predominantly employed RRMM as the primary approach, however, it remains uncertain whether patients will derive benefits from early-stage intervention strategies. Furthermore, the presence of mutant variants may differentially influence the response to currently approved or investigational TCE and CAR T-cell therapies. Therefore, future research should prioritize the utilization of longitudinal MM cell sequencing findings to facilitate the development and selection of targeted immunotherapy. Moreover, elucidating potential oncogenic dependency or molecular predisposition resulting from TNFRSF17 mutations or deletions would enable the optimization of early-stage strategies for immune therapy selection.

## Author contributions

CW: Conceptualization, Formal Analysis, Writing – original draft. WW: Data curation, Writing – original draft. MW: Data curation, Resources, Writing – original draft. JD: Data curation, Resources, Writing – original draft. CS: Data curation, Resources, Writing – original draft. YH: Writing – review & editing. SL: Supervision, Writing – review & editing.
